# Impeding Nucleation for More Significant Grain Refinement

**DOI:** 10.1038/s41598-020-66190-8

**Published:** 2020-06-10

**Authors:** Zhongyun Fan, Feng Gao, Bo Jiang, Zhongping Que

**Affiliations:** BCAST, Brunel University London, Uxbridge, Middlesex UB8 3PH UK

**Keywords:** Materials science, Structural materials

## Abstract

Grain refinement has been a topic of extensive research due to its scientific and technological importance as a common industrial practice for over seven decades. The traditional approach to grain refinement has been to reduce nucleation undercooling by the addition of potent nucleant particles. Here we show both theoretically and experimentally that more significant grain refinement can be achieved through increasing nucleation undercooling by using impotent nucleant particles. Based on the concept of explosive grain initiation, this new approach is illustrated by grain initiation maps and grain refinement maps and validated by experiments. It is anticipated that this new approach may lead to a profound change in both nucleation research and industrial practice well beyond metal casting.

## Introduction

Crystallization from liquids is a widespread phenomenon in both nature and technology, and has countless consequences in our everyday life^1,2^. For example, formation of ice in the atmosphere affects climate change^3^; controlling nucleation of molecular crystals from solutions is highly relevant to drug design and production^4^; protein crystal formation in living beings is responsible for many neurodegenerative disorders such as Alzheimer’s disease^5^; and grain refinement during solidification is critical for high performance engineering alloys^6^. In this paper we will focus our attention on grain formation during solidification of metallic materials.

Although nucleation plays a critical role in determining the solidified microstructure, it has been very much under-investigated due its associated experimental difficulties, with the majority of solidification research so far being concentrated on grain growth^7^. Classical homogeneous nucleation theory^8^ uses a thermodynamic approach to identify the critical cluster size and energy barrier, and deploys statistical mechanics to determine the nucleation rate^9–11^, rendering local fluctuation in atomic configuration, chemical composition and temperature extremely important. Based on this, heterogeneous nucleation on a substrate is facilitated by the liquid/substrate interface through reduction of the energy barrier for nucleation^12^. It is now generally accepted that nucleation in metallic systems is heterogeneous due to the inevitable existence of solid inclusions in metallic melts^13^. More recent advances in nucleation research include realization of the prenucleation phenomenon^14,15^, development of the epitaxial nucleation model based on structural templating^16^ and understanding the effect of substrate structure^15^, substrate chemistry^17^ and substrate surface roughness^18^ on heterogeneous nucleation.

Grain refinement during solidification processing is usually achieved by the addition of grain refiners (i.e., chemical inoculation)^19–21^. The traditional wisdom for grain refiner development is to search for the most potent solid particles practically available to reduce nucleation undercooling ($\Delta {T}_{n}$). The best example is the Al-5Ti-1B (all compositions are in wt.%) grain refiner that has been extensively researched and widely used in the metal casting industry^22^. Only until very recently it has been realized that what makes the Al-5Ti-1B grain refiner effective for grain refinement is the formation of an atomic monolayer of Al_3_Ti 2-dimensional compound (2DC) on the (0001) TiB_2_ surface that alters the lattice misfit with aluminum from −4.22% for TiB_2_ to 0.09% for TiB_2_ with Al_3_Ti-2DC, leading to a substantial increase in nucleation potency^23^. After more than seven decades of development by a trial-and-error approach, the commercial Al-5Ti-1B grain refiner is almost perfect in terms of particle size, size distribution and nucleation potency, leaving little space for further improvement^24^. However, it suffers from the “poisoning” effect in Al-alloys containing Zr^25,26^ or high levels of Si^27^. Another example is Mg-30Zr master alloy for refining Mg-alloys^28^, since Zr has the same crystal structure (hcp) and close-matching lattice parameters with Mg and thus a small lattice misfit (0.67% at 650 °C). Although used widely in industry it does not work for Al-containing Mg-alloys^29^. It is now high time we developed new approaches to achieve more significant grain refinement.

Another relevant advance in solidification research is the realization of the existence of an energy barrier for grain initiation after heterogeneous nucleation^30^. Nucleated solid particles can only grow freely once they reach a critical size to overcome the curvature constraint. The free growth model^30^ not only explains successfully why not all of the nucleant particles contribute to the formation of grains, but also provides a useful bridge between grain initiation events and the final grain size of the solidified microstructure^24^. This has triggered a substantial amount of effort for predicting the grain size of solidified microstructures (e.g., refs. ^31–33^).

In this paper, we investigate grain initiation behaviour and its effect on grain refinement through numerical simulation of solidification processes. Based on the simulation results, we have identified two distinctive grain initiation modes: progressive and explosive, which can be best presented by grain initiation maps and grain refinement maps. We show both theoretically and experimentally that more effective grain refinement can be achieved by nucleant particles with less nucleation potency.

### Grain initiation behaviour

**Figure 1:**
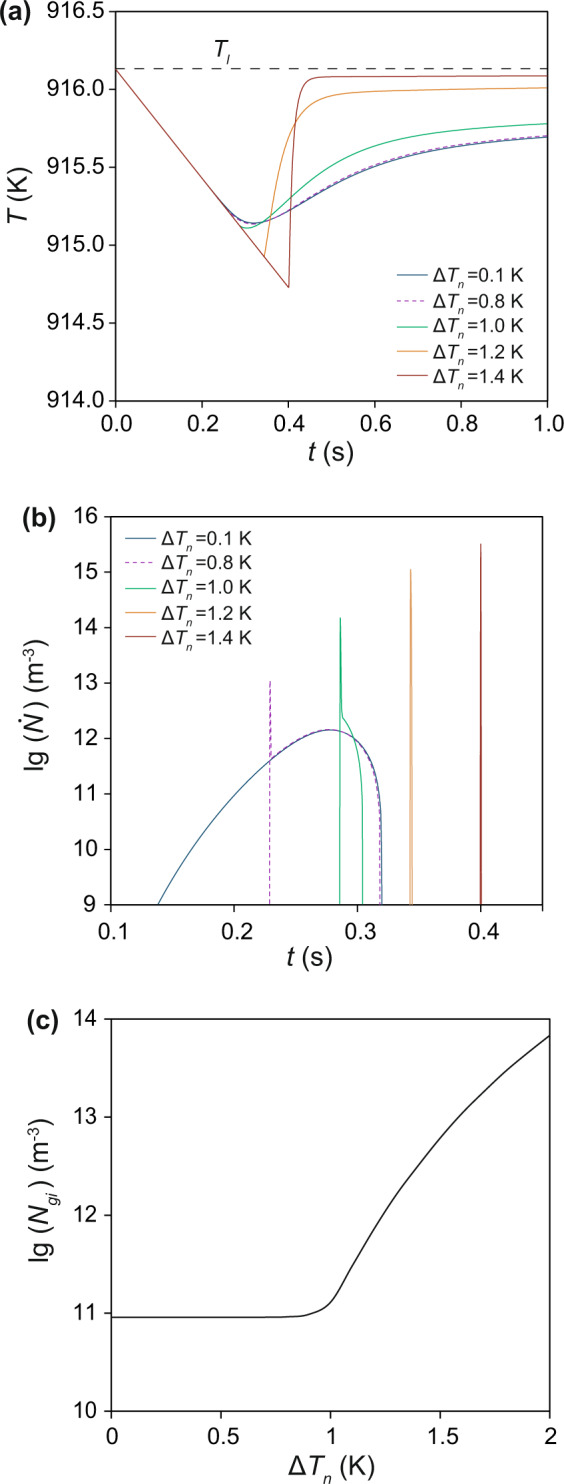
Solidification behaviour of Mg-1Al alloys containing nucleant particles of varying nucleation undercooling (from 0.1 K to 1.4 K). (**a**) Calculated cooling curves. (**b**) Calculated grain initiation rate as a function of time. (**c**) Initiated grain number density as function of nucleation undercooling. The nucleant particles are assumed to have a number density of 10^17^ m^−3^ and a log-normal size distribution (geometrical mean particle size *d*_0_ = 0.07 μm and standard deviation *σ* = 0.45).

Figure 1a shows the calculated cooling curves of Al-1Mg alloys inoculated by hypothetical nucleant particles, which have a varying nucleation undercooling ($\Delta {T}_{n}$), a fixed particle number density (${N}_{0}$, 10^17^ m^−3^) and a fixed log-normal size distribution (geometrical mean particle size *d*_0_ = 0.07 μm and standard deviation *σ* = 0.45). When $\Delta {T}_{n}$ = 0.1 K, the cooling curve exhibits a maximum undercooling ($\Delta {T}_{max}$) of 1.0 K; and an increase of $\Delta {T}_{n}$ to 0.8 K hardly changes the cooling curve at all. However, with a further increase of $\Delta {T}_{n}$ to 1.0 K, $\Delta {T}_{max}$ begins to increase, and reaches 1.2 K at $\Delta {T}_{n}=\Delta {T}_{max}$, which means that recalescence occurs at the nucleation temperature. When *ΔT*_*n*_ = 1.4 K, recalescence causes the temperature to rise almost instantaneously.

In order to understand such contrasting solidification behaviour in Fig. 1a, we have analyzed the evolution of grain initiation rate in each case, and the results are presented in Fig. 1b. When nucleant particles are potent (e.g., $\Delta {T}_{n}$ = 0.1 K), grain initiation occurs in a progressive manner over a period of 0.17 s; and the grain initiation rate initially increases with time, reaches a maximum at 0.14 s after grain initiation started, and decreases to 0 at the time of recalescence. When nucleant particles are moderately potent ($\Delta {T}_{n}$= 0.8 K or 1.0 K), we see an initial burst of grain initiation events that is followed by further grain initiation events in a progressive manner before recalescence. However, when nucleant particles are less potent ($\Delta {T}_{n}$ = 1.2 K and beyond), grain initiation takes place in an explosive manner within 10^−4^ s. Figure 1c shows the total grain initiation events (${N}_{gi}$) as a function of $\Delta {T}_{n}$. With increasing $\Delta {T}_{n}$, ${N}_{gi}$ is almost independent of $\Delta {T}_{n}$ at low $\Delta {T}_{n}$ and then increases sharply with increasing $\Delta {T}_{n}$ at high $\Delta {T}_{n}$. It becomes apparent from Fig. 1 that the difference in solidification behaviour manifested by the cooling curves in Fig. 1a is closely related to the variation of grain initiation behaviour, which changes from a progressive to an explosive manner with decreasing potency of nucleant particles.

**Figure 2:**
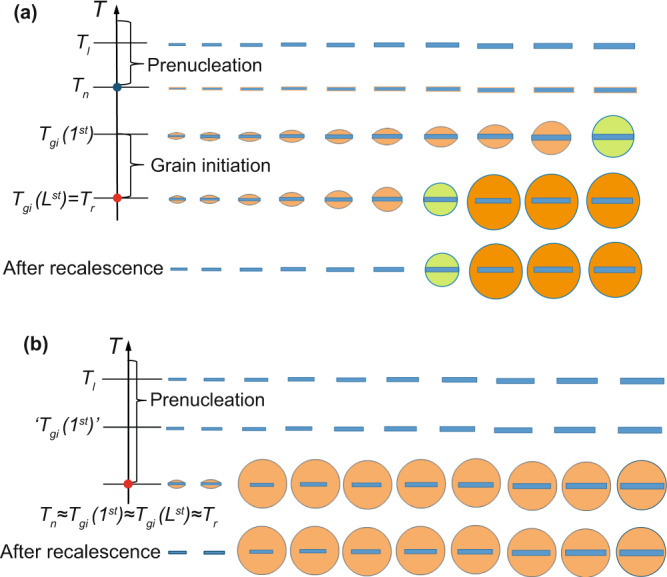
Schematic illustration of grain initiation behaviour during solidification of metallic alloys. (**a**) Progressive grain initiation process illustrated by solidification of alloys containing potent nucleant particles (e.g., Al-alloys inoculated by grain refiners containing TiB_2_ platelets). A prerequisite for progressive grain initiation is ${T}_{n} > {T}_{gi}({1}^{st})$. The first grain initiation event occurs on the largest solid particle at ${T}_{gi}({1}^{st})$; which is followed by grain initiation on progressively smaller solid particles; and the last grain initiation event occurs at ${T}_{gi}({L}^{st})={T}_{r}$. (**b**) Explosive grain initiation illustrated by solidification of alloys containing impotent nucleant particles. A prerequisite for explosive grain initiation is ${T}_{n} < {T}_{gi}({1}^{st})$. Immediately after nucleation at ${T}_{n}$, many solid particles are ready for grain initiation at the same time. In such cases, heterogeneous nucleation, grain initiation and recalescence all occur in an extremely short time interval; therefore, we have ${T}_{n}\approx {T}_{gi}({1}^{st})\approx {T}_{gi}({L}^{st})={T}_{r}$. After recalescence all the spherical caps that failed to initiate grains will dissolve back into the liquid.

Such distinctive grain initiation behaviour exhibited in Fig. 1b warrants further analysis. In the case of $\Delta {T}_{n}$= 0.1 K, heterogeneous nucleation takes place at the nucleation temperature (${T}_{n}$) on all available nucleant particles. With further increase of undercooling, the nucleated solid phase will grow and develop into spherical caps with a curvature dictated by the melt undercooling (Supplementary Eq. (1)), which is called constrained cap growth (see Supplementary Fig. 1). According to the grain initiation criterion^30^, the first grain initiation event takes place on the largest particle(s) of $d({1}^{{\rm{st}}})$ = 1.28 μm at a grain initiation undercooling of $\Delta {T}_{gi}({1}^{{\rm{st}}})$ = 0.46 K (Supplementary Fig. 2). This is followed by grain initiation events on progressively smaller particles; and the last grain initiation event occurs on particle(s) of $d({L}^{{\rm{st}}})$ = 0.6 μm at $\Delta {T}_{gi}({L}^{{\rm{st}}})=\Delta {T}_{max}$ = 1.0 K. Those spherical caps that failed to free-grow after recalescence will become thermodynamically unstable and will dissolve back into the melt during the subsequent solidification processes. The total number of grain initiation events is 9.09×10^10^ m^−3^ in this case. Such grain initiation behaviour is referred to as *progressive grain initiation* (PGI) as schematically depicted in Fig. 2a. A necessary condition for PGI is $\Delta {T}_{n} < \Delta {T}_{gi}({1}^{{\rm{st}}})$.

In the case of solidification of alloys that contain impotent particles (e.g. $\Delta {T}_{n}$ = 1.2 K), many nucleant particles (7.3 × 10^11^ m^−3^), with a particle size between 0.49 μm and 1.28 μm, satisfied the grain initiation criterion at undercoolings smaller than $\Delta {T}_{n}$. However, grain initiation can only occur after nucleation. Thus, in this case many solid particles are ready to free-grow almost simultaneously after nucleation, which causes an immediate recalescence, which in turn stifles any further grain initiation by the remaining solid particles with a smaller size. Similarly, those spherical caps that failed to free-grow will dissolve subsequently into the melt. The total number of grain initiation events in this case is 7.3 × 10^11^ m^−3^. Such grain initiation behaviour is referred to as *explosive grain initiation* (EGI), and is schematically depicted in Fig. 2b. A necessary condition for EGI is $\Delta {T}_{n}=\Delta {T}_{max}$.

### Grain initiation map

**Figure 3:**
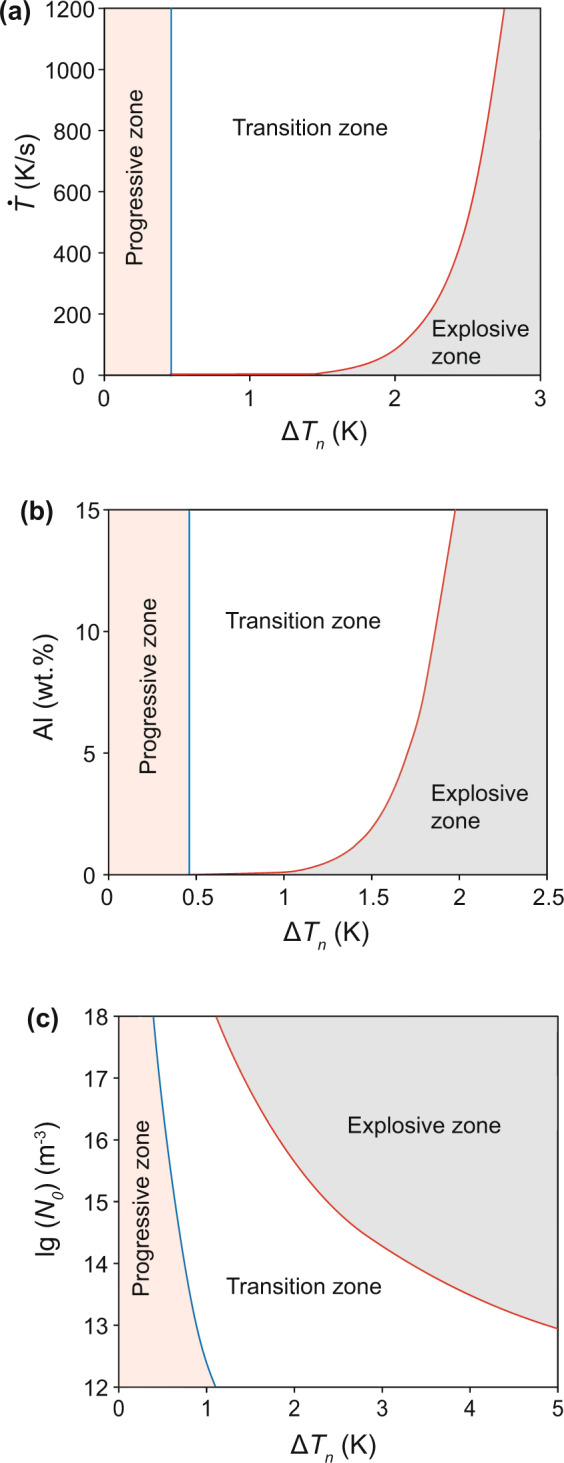
Grain initiation maps for Mg-Al alloys containing nucleant particles with varying nucleation potency but constant log-normal particle size distribution. (**a**) Grain initiation map ($\Delta {T}_{n}-\dot{T}$ plot) for Mg−1Al alloy with *N*_0_ = 10^17^ m^−3^ showing the effect of cooling rate on grain initiation behaviour. (**b**) Grain initiation map ($\Delta {T}_{n}-{C}_{0}$ plot) for Mg-Al alloys with *N*_0_ =10^17^ m^−3^ and $\dot{T}$ = 3.5 Ks^−1^ showing the effect of solute concentration on grain initiation behaviour. (**c**) Grain initiation map ($\Delta {T}_{n}-\,{\rm{lg}}({N}_{0})$ plot) for Mg-1Al alloy with $\dot{T}$ = 3.5 Ks^−1^ showing the effect of particle number density on grain initiation behaviour. The solid blue line marks the limit for progressive grain initiation ($\Delta {T}_{n}=\Delta {T}_{gi}({1}^{st})$) and red line marks the limit for explosive grain initiation ($\Delta {T}_{n}=\Delta {T}_{max}$). These two lines divide the grain initiation behaviour into 3 distinct zones: the progressive zone where $\Delta {T}_{n} < \Delta {T}_{gi}({1}^{st})$; the explosive zone $\Delta {T}_{n}=\Delta {T}_{max}$; and the transition zone where $\Delta {T}_{gi}({1}^{st})\le \Delta {T}_{n} < \Delta {T}_{max}$.

From the results in Fig. 1, it is apparent that there is a gradual transition from PGI to EGI with increasing $\Delta {T}_{n}$. This transition is demarcated by $\Delta {T}_{n}=\Delta {T}_{gi}({1}^{{\rm{st}}})$ for the start of the transition and $\Delta {T}_{n}=\Delta {T}_{gi}({L}^{{\rm{st}}})$ for the end of the transition. Both the start and the end of this transition can be determined numerically, and the results are presented in Fig. 3a, where the grain initiation behaviour is mapped in a $\Delta {T}_{n}-\dot{T}$ plot ($\dot{T}$ is cooling rate). The vertical blue line represents $\Delta {T}_{n}=\Delta {T}_{gi}({1}^{{\rm{st}}})$, and the curved red line represents $\Delta {T}_{n}=\Delta {T}_{gi}({L}^{{\rm{st}}})$. These two lines divide the $\Delta {T}_{n}$-$\dot{T}$ plot into 3 distinct zones: a PGI zone, an EGI zone and a transition zone.

Solidification in the PGI zone is characterized by $\Delta {T}_{n} < \Delta {T}_{gi}({1}^{{\rm{st}}})$. Heterogeneous nucleation takes place on all the nucleant particles; grain initiation starts with the largest solid particle(s) and then on progressively smaller ones until recalescence, which marks the last batch of grain initiation events with $\Delta {T}_{gi}({L}^{{\rm{st}}})=\Delta {T}_{max}$. In contrast, solidification in the EGI zone is characterized by *ΔT*_*n*_ = *ΔT*_*max*_. Immediately after heterogeneous nucleation, grain initiation takes place almost simultaneously on those solid particles that have satisfied the grain initiation criterion, and triggers instantaneous recalescence. Solidification in the transition zone is characterized by $\Delta {T}_{gi}({1}^{{\rm{st}}})\le \Delta {T}_{n}$<$\Delta {T}_{gi}({L}^{{\rm{st}}})$. Immediately after heterogeneous nucleation, grain initiation takes place simultaneously on those solid particles that satisfy the grain initiation criterion. However, latent heat released by such EGI events is insufficient to trigger recalescence; and further grain initiation can occur progressively on smaller solid particles until recalescence does occur. Grain initiation in the transition zone is in a mixed mode: explosive first and progressive later, as illustrated by the cases of $\Delta {T}_{n}$ = 0.8 K and $\Delta {T}_{n}$ = 1.0 K in Fig. 1b.

Similarly, grain initiation maps can be presented by a $\Delta {T}_{n}-{C}_{0}$ plot to illustrate the effect of solute concentration (${C}_{0}$) on grain initiation behaviour (Fig. 3b), or by a $\Delta {T}_{n}-{N}_{0}$ plot to illustrate the effect of particle number density (${N}_{0}$) on grain initiation behaviour (Fig. 3c).

The grain initiation maps in Fig. 3 reveal that PGI is favored by high nucleation potency (low $\Delta {T}_{n}$), high solute concentration, high cooling rate but low nucleant particle density, whilst EGI is favored by low nucleation potency, low solute concentration, low cooling rate but high nucleant particle density.

### Grain refinement map

**Figure 4:**
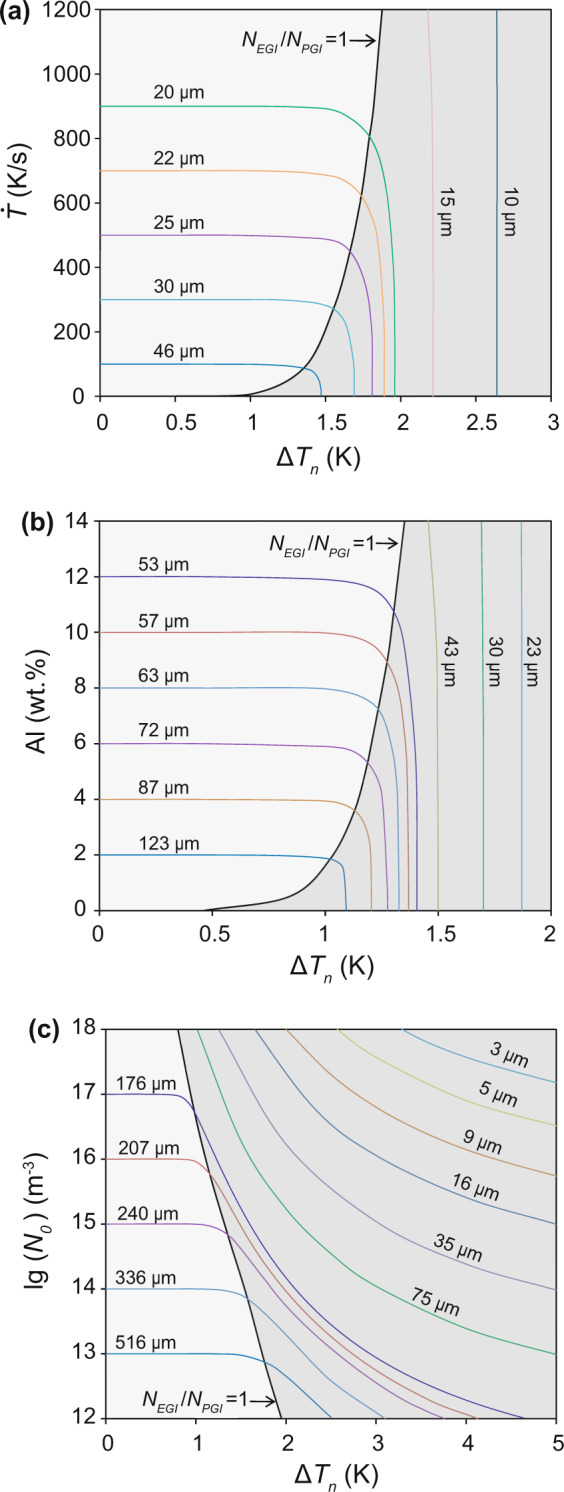
Grain refinement maps for Mg-Al alloys containing nucleant particles with varying nucleation potency but constant log-normal particle size distribution. (**a**) Grain refinement map ($\Delta {T}_{n}-\dot{T}$ plot) for Mg-1Al alloy with *N*_0_ = 10^17^ m^−3^ showing the effect of cooling rate on grain refinement. (**b**) Grain initiation map ($\Delta {T}_{n}-{C}_{0}$ plot) for Mg-Al alloys with *N*_0_ = 10^17^ m^−3^ and $\dot{T}$ = 3.5 Ks^−1^ showing the effect of solute concentration on grain refinement. (**c**) Grain initiation map ($\Delta {T}_{n}-lg({N}_{0})$ plot) for Mg-1Al alloy with $\dot{T}$ = 3.5 Ks^-1^ showing the effect of particle number density on grain refinement. The solid black line represents the conditions where explosive grain initiation has equal proportion with progressive grain initiation; the light and dark grey coloured zones mark PGI-dominant and EGI-dominant zones, respectively.

To understand the effect of grain initiation behaviour on grain refinement we have developed the concept of the grain refinement map. The average grain size can be calculated by grain number density (Supplementary Eq. (10)). Figure 4a shows iso-grain-size lines in a $\Delta {T}_{n}-\dot{T}$ plot. Also shown in Fig. 4a is a solid black line that represents ${N}_{PGI}:{N}_{EGI}$ = 1, where ${N}_{PGI}$ and ${N}_{EGI}$ are the number of PGI and EGI events, respectively. This solid line separates the entire area in Fig. 4a into two distinct zones: the PGI-dominant zone (${N}_{PGI} > {N}_{EGI}$) and the EGI-dominant zone (*N*_*EGI*_ > *N*_*PGI*_). In the PGI-dominant zone, grain size decreases with increasing cooling rate, but is almost independent of $\Delta {T}_{n}$ for a given cooing rate, whilst in the EGI-dominant zone, grain size decreases with increasing $\Delta {T}_{n}$, but is almost independent of cooling rate for a given $\Delta {T}_{n}$.

Similarly, the grain refinement map can be represented as a $\Delta {T}_{n}-{C}_{0}$ plot (Fig. 4b). It is clear from Fig. 4b that in the PGI-dominant zone, grain size decreases moderately with increasing ${C}_{0}$ and is independent of $\Delta {T}_{n}$ for a given alloy composition, whilst in the EGI-dominant zone, grain size decreases sharply with increasing $\Delta {T}_{n}$ and is almost independent of solute concentration for a given $\Delta {T}_{n}$. Finally, the grain refinement map is shown in Fig. 4c as a $\Delta {T}_{n}-{N}_{0}$ plot. Figure 4c reveals that in the PGI-dominant zone, grain size decreases moderately with increasing ${N}_{0}$, whilst in the EGI-dominant zone, grain size decreases with not only increasing ${N}_{0}$ but also increasing $\Delta {T}_{n}$.

**Figure 5:**
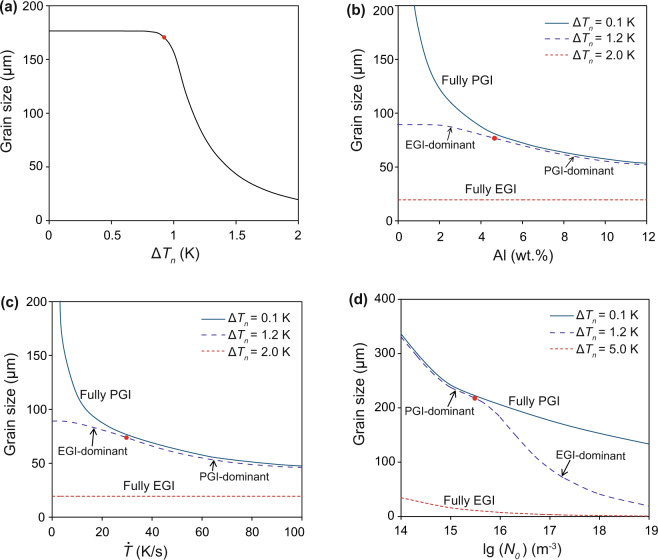
The effect of solidification conditions on grain size of Mg-Al alloy. The effect of potency of (**a**) nucleant particles, (**b**) solute concentration, (**c**) cooling rate, and (**d**) particle number density with the same log-normal distribution (*d*_0_ = 0.07 μm and *σ* = 0.45). Other specific conditions for the calculations are: Mg-1Al alloy and *N*_0_ = 10^17^ m^−3^, $\dot{T}$ = 3.5 Ks^−1^ for (**a**); *N*_0_ = 10^17^ m^−3^, $\dot{T}$ = 3.5 Ks^−1^ for (**b**); Mg-1Al alloy and *N*_0_ = 10^17^ m^−3^ for (**c**); Mg-1Al alloy and $\dot{T}$ = 3.5 Ks^−1^ for (**d**). The red point represents the conditions for which 50% of the total number of grains are initiated explosively.

Based on the grain refinement maps in Fig. 4, we now analyze quantitatively the effect of $\Delta {T}_{n},\,{C}_{0}$ and ${N}_{0}$ on grain size. Figure 5a shows the grain size of Mg-1Al alloy solidified under a cooling rate of 3.5 Ks^−1^ inoculated with nucleant particles with a constant number density and size distribution but with varying nucleation potency. Figure 5a suggests that when PGI is dominant, grain size is independent of $\Delta {T}_{n}$; while when EGI is dominant, grain size decreases sharply with increasing $\Delta {T}_{n}$. Under such conditions there is no further grain refinement by inoculation with more potent nucleant particles as long as PGI is dominant; further grain refinement can only be achieved by promotion of EGI using less potent nucleant particles.

Figure 5b shows the effect of ${C}_{0}$ on grain refinement with varying grain initiation behaviour. When nucleation potency is high (e.g., $\Delta {T}_{n}=0.1\,{\rm{K}}$), grain initiation is fully progressive, resulting in an overall large grain size that decreases with increasing ${C}_{0}$. When the nucleation potency is moderate (e.g., $\Delta {T}_{n}=1.2\,{\rm{K}}$), there is a transition of grain initiation behaviour from EGI-dominant to PGI-dominant with increasing ${C}_{0}$. In this case, ${C}_{0}$ has only little effect on grain size. In contrast, when nucleation potency is low (e.g., $\Delta {T}_{n}=2.0\,{\rm{K}}$), grain initiation is fully explosive, and grain size is constantly small and is independent of *C*_0_. The effect of cooling rate on grain refinement has a similar trend to that of the composition, as shown in Fig. 5c.

We use Fig. 5d to illustrate the effect of particle number density on grain refinement. When grain initiation is fully progressive, an increase in ${N}_{0}$ leads to a moderate decrease in grain size. More significant grain refinement can only be achieved by promotion of EGI through increasing ${N}_{0}$ and $\Delta {T}_{n}$. However, when grain initiation is fully explosive, grain size becomes consistently small and has little dependence on ${N}_{0}$.

To sum up, from the grain refinement maps in Figs. 4 and 5 the following conclusions can be drawn:

- For a given alloy solidifying under a given cooling rate, grain refinement is favored by inoculation with more impotent nucleant particles (large $\Delta {T}_{n}$) with a constant particle number density.
- When progressive grain initiation is dominant, grain refinement is promoted by increasing cooling rate, solute concentration and number density of nucleant particles, but is almost independent of the potency of nucleant particles.
- When explosive grain initiation is dominant, grain refinement is favored by decreasing the potency of nucleant particles and increasing number density of nucleant particles, but is largely independent of both cooling rate and solute concentration.

### Experimental validation

**Figure 6:**
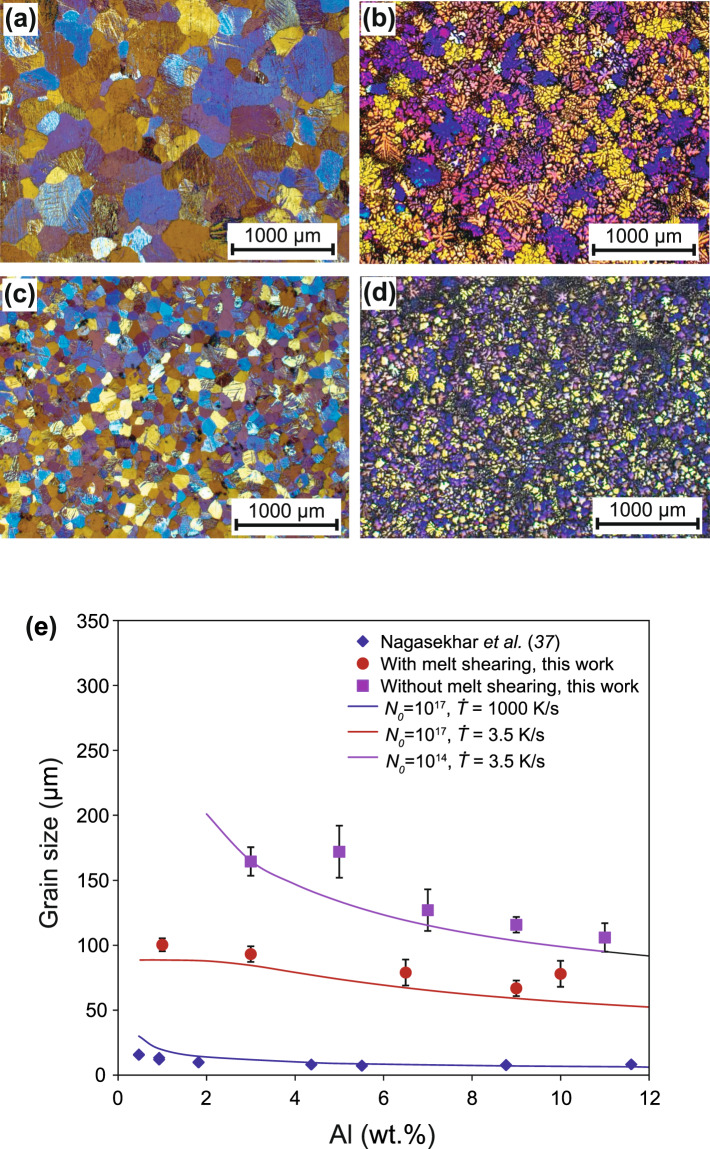
Experimental validation of theoretical predictions of grain size. Optical micrographs showing the microstructures of TP-1 test samples of Mg-3Al **(a, c)** and Mg-9Al **(b, d)** solidified without prior melt shearing (**a, b**) with prior melt shearing (**c, d**). **(e)** Theoretically predicted grain size compared with the experimentally measured grain size of Mg-Al alloys solidified under different solidification conditions. The good agreement between the theoretical predictions and the experimental grain size data (with two orders of magnitude variation) produced under 3 orders of magnitude change in cooling rate provides a strong validation of the theoretical model presented in this paper.

Figure 6a–d are optical micrographs showing the microstructures of Mg-3Al and Mg-9Al alloys solidified with and without prior intensive melt shearing (a technique for dispersing solid particles in a liquid^34^) and solidified in a TP-1 mold with $\dot{T}$ = 3.5 Ks^−1 ^^35^. Figures 6a–d show that grain size with intensive melt shearing decreases significantly, while an increase in solute concentration only has a moderate effect on grain size. These results can utilized to validate the theory presented in this paper.

Grain refinement is a complex process and its outcome depends strongly on the interplay between heterogeneous nucleation and grain initiation processes. For Mg-Al melt without addition of grain refiner the nucleating particles are most likely the native MgO particles, which have a lattice misfit with Mg of 8.19%^34^ and a $\Delta {T}_{n}$ = 1.2 K. When the Mg-3Al alloys are solidified in the TP-1 mold without prior melt shearing, the estimated MgO particle density is 10^14^ m^−3 ^^36^. In this case, the grain initiation behaviour is predominantly progressive (Fig. 4c for low Al content), resulting in a grain size of 164 μm. With prior melt shearing, the estimated MgO particle density is increased up to 10^17^ m^−3 ^^36^. This increase in nucleant particle number density changes the grain initiation behaviour from PGI-dominant to EGI-dominant (Fig. 4c) and leads to a decrease in grain size to 93 μm. When the Al content is increased from 3 wt.% to 9 wt.% with prior melt shearing, the grain size is decreased slightly to 67 μm due to the increasing growth restriction of solute.

The experimentally determined grain size of Mg-Al alloys solidified under different conditions is compared with theoretical predictions in Fig. 6e. The good agreement between theoretical predictions and experimental results provides a direct evidence of the validity of the present theory. In addition, high pressure die casting of Mg-Al-alloys with a cooling rate in the order of 10^3^ Ks^−1^ and a high shear rate (10^5^/s) at the gate leads to a more significant grain refinement (Fig. 4a), with the grain size being a few microns (Fig. 6e) and in good agreement with the experimental data in the literature^37^. Furthermore, Fig. 6e also confirms the theoretical prediction that solute concentration has a moderate effect on grain size when PGI is dominant and becomes increasingly less important when grain initiation become increasingly explosive. The good agreement in Fig. 6e between the theoretical predictions and the experimental grain size data (with two orders of magnitude variation) produced under 3 orders of magnitude change in cooling rate provides a strong support to the theoretical model presented in this paper.

## Discussions

Heterogeneous nucleation refers to the process of creating on a substrate, a crystalline template from which the new solid phase can grow in the liquid. As an atomistic process heterogeneous nucleation should occur on all available nucleant particles and be independent of the substrate size. According to the epitaxial nucleation model^16^, heterogeneous nucleation starts at a critical undercooling ($\Delta {T}_{n}$) and proceeds layer-by-layer through a structural templating mechanism. $\Delta {T}_{n}$ can be taken as a measure of nucleation potency, which is affected by lattice misfit between the substrate and the solid phase^15^, chemical interaction between the substrate and the liquid^17^ and atomic level surface roughness of the substrate^18^.

Grain initiation refers to the process in which a solid particle of the new phase can grow freely to become a grain in the solidified microstructure (Fig. 2). Compared with heterogeneous nucleation, grain initiation occurs at a much later time and a much larger length scale. Grain initiation has so far been treated in a progressive manner in the literature^30^. In this paper we have introduced explosive grain initiation as a new grain initiation mode. Different from PGI, EGI is promoted by low cooling rate, low solute concentration, high nucleation undercooling and high particle number density (Fig. 3). In practical cases, EGI should lead to more significant grain refinement than PGI (Fig. 4).

For grain refinement, the conventional wisdom is to *decrease the nucleation undercooling* by addition of grain refiners that contain potent nucleant particles (i.e., more potent than the native particles). The best example of such an approach is the Al-Ti-B based grain refiners^6,19–21^. TiB_2_ particles in an appropriately prepared Al-5Ti-1B grain refiner have a monolayer of Al_3_Ti-2DC on their (0001) surface^23^, which has a lattice misfit of 0.09% with α-Al. This makes the TiB_2_/Al_3_Ti-2DC particles extremely potent for heterogeneous nucleation of α-Al ($\Delta {T}_{n}$ is extremely small^38^), and the grain initiation is fully progressive (Fig. 3), resulting in a grain size of a few hundreds of microns^24^. The concept of EGI provides a new approach to grain refinement by increasing $\Delta {T}_{n}$, which has the potential to push grain refinement to a new level unachievable by the conventional approach.

This research suggests that native solid particles available in alloy melts can be used for more effective grain refinement than chemical inoculation. Mg-alloys provide us with the best alloy system to implement this new approach. As the only solid species of significance in Mg alloy melts, MgO has a large lattice misfit with α-Mg (8.19%), a very small particle size (50–200 nm) and a large number density (10^17^ m^−3^). This makes MgO very impotent for heterogeneous nucleation but particularly effective for grain refinement through promotion of EGI. Extensive melt shearing leads to a significant increase in MgO number density, which in turn promotes EGI during solidification and offers significant grain refinement (Fig. 6e).

It should be pointed out that grain refinement by native nucleant particles is more advantageous than chemical inoculation from the view point of closed-loop recycling. Chemical inoculation is very inefficient since only a small fraction of the added nucleant particles (<1%) are effective for grain initiation^24^; and the majority of the added particles end up in the inter-dendritic regions of the solidified microstructure and have an adverse effect on the mechanical performance. In addition, accumulation of such inoculant particles represents a severe contamination in the recycled alloys, and makes recycling increasingly more difficult.

Heterogeneous nucleation is a wide spread phenomenon in both nature and technology^1^. Condensation and evaporation, grain growth, deposition of thin films and overall crystallization are but a few of the processes in which nucleation plays a prominent role^2^. Our newly proposed concepts of explosive grain initiation, the grain initiation map and the grain refinement map are highly relevant or even directly applicable to such wider scientific and technological research fields.

## Summary

We have used a numerical approach to investigate the grain initiation process during solidification of metallic alloys under a variety of solidification conditions. Based on our simulation results we have identified two distinct grain initiation modes: progressive and explosive. Progressive grain initiation starts with the largest solid particle(s), continues with progressively smaller ones and finishes at recalescence; whilst during explosive grain initiation many solid particles initiate grains almost simultaneously and cause an immediate recalescence. Grain initiation maps have been developed to describe the effects of potency of nucleant particles, solute concentration, cooling rate and nucleant particle number density on grain initiation behaviour. Further analysis of the effect of grain initiation behaviour on grain refinement has led to the development of grain refinement maps. The traditional wisdom for grain refinement is to decrease nucleation undercooling by addition of potent nucleant particles, as exemplified by the commercial Al-5Ti-1B grain refiner. In this work we have shown both theoretically and experimentally that more effective grain refinement can be achieved by increasing nucleation undercooling using less potent nucleant particles. Since heterogeneous nucleation is a wide spread phenomenon in both nature and technology, it is anticipated that this new approach may lead to a profound change in both nucleation research and industrial practice well beyond metal casting.

## Methods

### Method for numerical simulation

We have numerically modelled the key solidification processes (see Supplementary). A Matlab code was compiled to perform the numerical simulation. All the parameters used as input for the numerical simulations are summarized in Supplementary Table 1. The total volume used for all the simulations is fixed at 1 cm^3^. The time step used is usually 10^−3^ s but 10^−5^ s in cases of explosive grain initiation. In addition, we have used other dendrite growth models, such as the LKT model^39^, to calculate dendritic growth velocity ${v}_{d}$, and found that the simulation results are insensitive to the dendritic growth model used.

To simplify numerical modelling, we have made the following basic assumptions:

- The alloy melt is in an isothermal condition.
- The heat extraction rate by the environment is constant.
- Morphological instability occurs when the spherical growth velocity equals the dendritic growth velocity.
- The size of the nucleant particles has a log-normal distribution.

### Method for experimental validation

To validate theoretical predictions, commercial purity Mg (CP-Mg: Mg-0.04Al-0.02Mn-0.013Si-0.002Fe-0.001Cu) and high purity Al (99.99%) were used for preparing the Mg-Al alloys studied in this work. All alloy melts were prepared in an electric furnace (set at 680 °C) with a steel crucible, under the protection of a gas mixture of N_2_ + 0.5vol.% SF_6_. Melt shearing was conducted using a rotor-stator high shear device^34^ at 680 °C for 10 minutes with a rotation speed of 5000 rpm. Alloy melts with 10 K superheat were cast into a standard TP-1 mold (preheated to 350 °C) for solidification at a nominal cooling rate of 3.5 Ks^−1 ^^35^. Grain size was assessed on a polished and colour-etched cross-section 38 mm above the base of the TP-1 sample using a standard intercept length method.

## Supplementary information

Supplementary Information.

## Data Availability

All data in the main textand the supplementary and the custom Matlab codes are available from the corresponding author on reasonable request.
