# Supplementary material for: Impeding Nucleation for More Significant Grain Refinement
Source: Sci Rep. 2020 Jun 10;10:9448. doi: 10.1038/s41598-020-66190-8 (PMC7287079; doi:10.1038/s41598-020-66190-8)
Supplement: Supplementary file 1 — Supplementary Information. [file 41598_2020_66190_MOESM1_ESM.pdf]

Supplementary information for

Impeding Nucleation for More Significant Grain Refinement

Zhongyun Fan*, Feng Gao, Bo Jiang, Zhongping Que

BCAST, Brunel University London, Uxbridge, Middlesex, UB8 3PH, UK.

*Correspondence to: zhongyun.fan@brunel.ac.uk

Solidification processes

Based on the recent advances in the understanding of heterogeneous nucleation[1-5] and our current knowledge of grain growth[6], solidification of a single phase alloy may involve a number of individual stages, as schematically illustrated in **Supplementary Fig. 1**. Here we analyze briefly these stages to establish a physical model of solidification to facilitate numerical modelling.

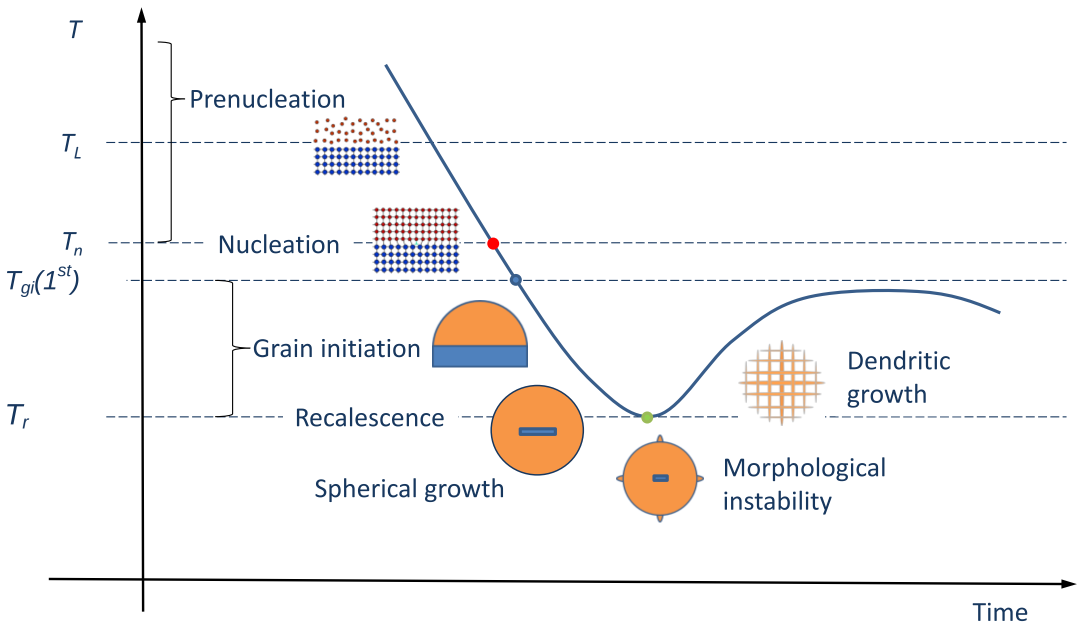

Supplementary Fig. 1. A schematic cooling curve illustrating the stages of the solidification process by following a specific nucleant particle (in blue) that initiates a grain in the solidified microstructure. Please note that the length scale of the sketches increases with time during the solidification process.

Prenucleation refers to the phenomenon of pronounced atomic ordering in the liquid adjacent to the substrate/liquid interface at temperatures above the nucleation temperature (T_n)[2]. Prenucleation manifests itself by substantial atomic layering along the normal of the substrate surface and in-plane ordering parallel to the substrate surface[1,2]. Such atomic ordering is affected by the lattice misfit between the substrate and the new solid phase[2], chemical interaction between atoms of the substrate and the liquid[3] and atomic-level surface roughness of the substrate[5]. Therefore, prenucleation produces a precursor for the subsequent heterogeneous nucleation process.

Heterogeneous nucleation creates a template on a substrate in the liquid, from which the new solid phase (either stable or metastable) can grow. It occurs at a nucleation temperature, T_n, through structural templating by the substrate and proceeds by a layer-by-layer growth mechanism, as described in the epitaxial nucleation model[3]. Epitaxial nucleation is independent of the size of nucleant particles; once the nucleation undercooling is achieved,

heterogeneous nucleation should take place on all nucleant particles[3]. However, a nucleus formed at this stage may not necessarily lead to a grain in the solidified microstructure.

Constrained cap growth refers to the initial growth process experienced by a nucleus before grain initiation. After nucleation, the solid phase cannot grow at a constant temperature due to the constraint of the curvature effect. Further growth can only be possible by increasing the undercooling to overcome the curvature constraint. Such constrained cap growth of a solid particle continues until the grain initiation undercooling is reached[7].

Grain initiation refers to the process of formation of a grain of radius (r), which can grow freely with a grain initiation undercooling (ΔT_{gi}). r can be understood to be the radius of the smallest sphere of the solid phase that engulfs the entire nucleant particle, and thus r equals the maximum dimension of the nucleant particle. Grain initiation is governed by the grain initiation criterion[7]:

$$\Delta T_{gi} = \frac{2\Gamma}{r}, \tag{1}$$

where Γ is the Gibbs-Thompson coefficient. Eq. (1) suggests that grain initiation starts with the largest solid particle and the on progressively smaller ones. Grain initiation is a deterministic process and is dependent on the size of the solid particle[7]. It is clear that not every nucleus formed at T_n can grow freely to initiate a grain. Those that fail to grow freely will become thermodynamically unstable and will simply dissolve back into the liquid.

Spherical growth occurs immediately after grain initiation with a growth velocity of v_s[8]:

$$v_s = \frac{\alpha}{1-\alpha}\left(1+\sqrt{\alpha}+\alpha\right)\frac{D}{r'}, \tag{2}$$

where r' is the radius of the solid particle, D is the diffusion coefficient of the solute, and α is the supersaturation defined by Zener[9], which can be calculated by the following equation[8]:

$$\alpha = \frac{\Delta T}{(mC_0-\Delta T)(k-1)}, \tag{3}$$

where m is the liquidus slope, C_0 is the solute concentration, k is the solute partition coefficient and ΔT is the growth undercooling.

Dendritic growth: At a critical size, the spherical morphology becomes unstable[10] and a grain starts to grow dendritically with a growth velocity of v_d[11]:

$$v_d = \frac{D}{2\Gamma}\left\{\frac{\Delta T}{a_0[mC_0(k-1)]^{a_1}}\right\}^{\frac{1}{1-a_1}}, \tag{4}$$

where $a_0 = 2.08 - 1.534\text{k} - 0.434k^2$, and $a_1 = 0.648 + 0.124k$.

Recalescence occurs at a temperature, T_r (the recalescence temperature), at which the heat released by solidification equals the heat dissipated into the environment. After recalescence the rising temperature (i.e., decreased undercooling) will stifle any further grain initiation. Therefore, it is usually assumed that the number of grains in the final solidified microstructure equals that already initiated at recalescence[7,12-14].

Method for numerical simulation

We have numerically modelled the key solidification processes described in the previous section. At each stage, the heat extracted from the melt to the environment (Q_E) equals to the heat released due to temperature drop and/or the latent heat (Q_R):

$$\frac{dQ_E}{dt} = \frac{dQ_R}{dt}. \tag{5}$$

When $t < t_n$ where t_n is the time at which nucleation occurs, the heat released is only due to the temperature drop and one has

$$\frac{dQ_R}{dt} = c_{pv} \frac{dT}{dt}, \tag{6}$$

where c_{pv} is the specific heat capacity of the melt per unit volume.

When $t_n \leq t \leq t_r$ where t_r is the time at which recalescence takes place, the heat released is due to both temperature drop and latent heat and one has

$$\frac{dQ_R}{dt} = \left(c_{pv} + \Delta H_v \frac{df_s}{dT}\right) \frac{dT}{dt}, \tag{7}$$

where ΔH_v is the latent heat of fusion per unit volume, and f_s is the fraction of the solid phase.

When $t > t_r$, no further grain initiation occurs, and the heat balance also follows Eq. (7).

As revealed by experimental observation[15,16], nucleant particles have a log-normal size distribution:

$$\frac{dN_d}{dd} = \frac{N_0}{\sigma d \sqrt{2\pi}} exp - \left(\frac{[ln(d) - ln(d_0)]^2}{2\sigma^2}\right), \tag{8}$$

where N_0 is the total number density of the particles in the melt, N_d is the number density of the particles of size d, d_0 is the geometrical mean particle size, and σ is the standard deviation of the particle size distribution. Eq. (8) is the probability distribution function. Using the cumulative distribution function, the number of nucleant particles that have diameters larger than d can be calculated:

$$\int_d^{+\infty} dN_d = N_0 \left[\frac{1}{2} - \frac{1}{2} erf\left(\frac{ln\ d - ln\ d_0}{\sigma\sqrt{2}}\right)\right], \tag{9}$$

where erf is the errorfunction.

To conduct a numerical simulation we need to determine the size of the largest particle, $d(1^{\text{st}})$, which has a cumulative distribution function of 1 and a grain initiation undercooling, $\Delta T_{gi}(1^{\text{st}})$. The results for the Mg-1Al/MgO system are presented in **Supplementary Fig. 2**. It should be pointed out that both $d(1^{\text{st}})$ and $\Delta T_{gi}(1^{\text{st}})$ are functions of d_0, σ, N_0 and the total volume (assumed here to be 1 cm^3). For a given type of nucleant particle with log-normal size distribution, $d(1^{\text{st}})$ increases and $\Delta T_{gi}(1^{\text{st}})$ decreases with increasing particle number density.

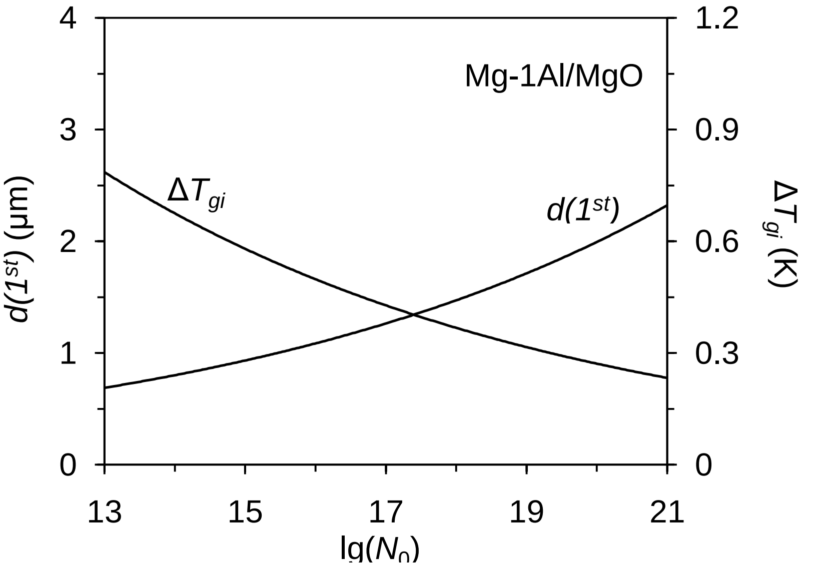

Supplementary Fig. 2. Calculated size of the largest nucleant particle ($d(1^{st})$) and its corresponding grain initiation undercooling $\Delta T_{gi}(1^{st})$ as a function of total number density of nucleant particles (N_0, m^{-3}) for Mg-1Al alloy containing native MgO particles.

It has been confirmed by experiment that in the fully dispersed state MgO particles in Mg melts have a log-normal size distribution with $d_0 = 0.07$ μm, $\sigma = 0.45$ and $N_0 = 10^{17}$ m^{-3} [16]. MgO particles in Mg-alloys has been used as a reference point for the nucleant particles in this study.

The average grain size (d_G) is calculated from the number of grains per unit volume which is assumed to be equal to the total number density of initiated grains (N_{gi}) [7]:

$$d_G = \left(\frac{0.5}{N_{gi}}\right)^{1/3}. \qquad (10)$$

To simplify numerical modelling, we have made the following basic assumptions:

- The alloy melt is in an isothermal condition.
- The heat extraction rate by the environment is constant.
- Morphological instability occurs at $v_d = v_s$.
- The size of the nucleant particles has a log-normal distribution.

A matlab code was compiled to perform the numerical simulation. All the parameters used as input for the numerical simulations are summarized in **Supplementary Table 1**. The total volume used for all the simulations is fixed at 1 cm^3. The time step used is usually 10^{-3} s but 10^{-5} s in cases of explosive grain initiation. In addition, we have used other dendrite growth models, such as the LKT model[17], to calculate v_d, and found that the simulation results are insensitive to the dendritic growth model used.

Supplementary Table 1. Values for physical parameters used in numerical simulations.

**Parameters (symbol, unit)**	**Mg-Al/MgO system**
Partition coefficient (k)	0.37 [18]
Liquidus slope (m, K(wt%)$^{-1}$)	-6.87 [18]
Specific heat capacity (c_{pv}, Jm^{-3}K^{-1})	2.59×10^{6} [19]
Enthalpy of fusion (ΔH_V, Jm^{-3})	6.75×10^{8} [19]
Diffusion coefficient of solute (D, m^{2}s^{-1})	2.7×10^{-9} [19]
Gibbs-Thompson coefficient (Γ, Km)	1.48×10^{-7} [19]
Mean particle size of log-normal distribution mean (d_0, m)	7.0×10^{-8} [16]
Standard deviation of log-normal distribution (σ)	0.45 [16]

References

1. Men, H. & Fan, Z. Atomic ordering in liquid aluminium induced by substrates with misfits. *Comput. Mat. Sci.* **85**, 1-7 (2014).

2. Men, H. & Fan, Z. Prenucleation induced by crystalline substrates. *Metall. Mater. Trans. A* **49**, 2766-2777 (2018).

3. Fan, Z. An epitaxial model for heterogeneous nucleation on potent substrates. *Metall. Mater. Trans. A* **44**, 1409-1418 (2013).

4. Fang, C. M., Men, H. & Fan, Z. Effect of substrate chemistry on prenucleation. *Metall. Mater. Trans. A* **49**, 6231-6242 (2018).

5. Jiang, B., Men, H. & Fan, Z. Atomic ordering in the liquid adjacent to an atomically rough solid surface. *Comput. Mat. Sci.***153**, 73-81 (2018).

6. Dantzig, J., Rappaz, M., Eds., *Solidification (Engineering Sciences: Materials)* (CRC Press, Lausanne, 2009).

7. Greer, A. L., Bunn, A. M., Tronche, A., Evans, P. V. & Bristow, D. J. Modelling of inoculation of metallic melts: application to crystal refinement of aluminium by Al-Ti-B. *Acta Mater.* **48**, 2823-2835 (2000).

8. Fan, Z. & Lu, S. Z. A simple model for spherical growth in alloy solidification. *IOP Conf. Series: Materials Science and Engineering* **117**, 012016 (2016).

9. Zener, C. Theory of growth of spherical precipitates from solid solution. *J. Appl. Phys.* **20**, 950-953 (1949).

10. Mullins, W. W. & Sekerka, R. F. Morphological stability of a particle growing by diffusion or heat flow. *J. Appl. Phys.* **34**, 323-329 (1963).

11. Hunt, J. D. & Fan, Z. A simple model for the nucleation and growth of equiaxed grains. *Proceedings of the John Hunt Inter. Symposium* (2011) 59-78.

12. Shu, D., Sun, B., Mi, J. & Grant, P. S. A quantitative study of solute diffusion field effects on heterogeneous nucleation and the crystal size of alloys. *Acta Mater.* **59**, 2135-2144 (2011).

13. StJohn, D. H., Qian, M., Easton, M. A. & Cao, P. The interdependence theory: the relationship between crystal formation and nucleant selection. *Acta Mater.* **59**, 4907-4921 (2011).

14. Du, Q. & Li, Y. An extension of the Kampmann-Wagner numerical model towards as-cast crystal size prediction of multicomponent aluminum alloys. *Acta Mater.* **71**, 380-389 (2014).

15. Quested, T. E. & Greer, A. L. The effect of the size distribution of inoculant particles on as-cast crystal size in aluminium alloys. *Acta Mater.* **52**, 3859-3868 (2004).

16. Men, H., Jiang B. & Fan, Z. Mechanisms of grain refinement by intensive shearing of AZ91 alloy melt. *Acta Mater.* **58**, 6526-6534 (2010).

17. Lipton, J., Kurz, W. & Trivedi, R. Rapid dendrite growth in undercooled alloys. *Acta Metall.* **35**, 957-964 (1987).

18. StJohn, D. H., Qian, M., Easton, M. A., Cao, P. & Hildebrand, Z. Crystal refinement of magnesium alloys. *Metall. Mater. Trans. A* **36**, 1669-1679 (2005).

19. Avedesian, M. & Baker, H. in *Magnesium and Magnesium Alloys*, M. Avedesian, H. Baker Eds., (ASM International, Materials Park, OH, 1999).
